# Advanced deep transfer learning techniques for efficient detection of cotton plant diseases

**DOI:** 10.3389/fpls.2024.1441117

**Published:** 2024-12-19

**Authors:** Prashant Johri, SeongKi Kim, Kumud Dixit, Prakhar Sharma, Barkha Kakkar, Yogesh Kumar, Jana Shafi, Muhammad Fazal Ijaz

**Affiliations:** ^1^ School of Computer Science and Engineering, Galgotias University, Greater Noida, India; ^2^ Department of Computer Engineering, Chosun University, Gwangju, Republic of Korea; ^3^ Department of Computer Application, Dharma Samaj (D.S.) College, Aligarh, India; ^4^ Department of Information Technology (IT), Institute of Technology and Science, Mohan Nagar Ghaziabad, India; ^5^ Department of Computer Science and Engineering (CSE), School of Technology, Pandit Deendayal Energy University, Gandhinagar, Gujarat, India; ^6^ Department of Computer Engineering and Information, College of Engineering in Wadi Alddawasir, Prince Sattam Bin Abdulaziz University, Wadi Alddawasir, Saudi Arabia; ^7^ School of Information Technology (IT) and Engineering, Melbourne Institute of Technology, Melbourne, VIC, Australia

**Keywords:** cotton disease, agriculture, deep learning, bacterial blight, powdery mildew, contour features

## Abstract

**Introduction:**

Cotton, being a crucial cash crop globally, faces significant challenges due to multiple diseases that adversely affect its quality and yield. To identify such diseases is very important for the implementation of effective management strategies for sustainable agriculture. Image recognition plays an important role for the timely and accurate identification of diseases in cotton plants as it allows farmers to implement effective interventions and optimize resource allocation. Additionally, deep learning has begun as a powerful technique for to detect diseases in crops using images. Hence, the significance of this work lies in its potential to mitigate the impact of these diseases, which cause significant damage to the cotton and decrease fibre quality and promote sustainable agricultural practices.

**Methods:**

This paper investigates the role of deep transfer learning techniques such as EfficientNet models, Xception, ResNet models, Inception, VGG, DenseNet, MobileNet, and InceptionResNet for cotton plant disease detection. A complete dataset of infected cotton plants having diseases like Bacterial Blight, Target Spot, Powdery Mildew, Aphids, and Army Worm along with the healthy ones is used. After pre-processing the images of the dataset, their region of interest is obtained by applying feature extraction techniques such as the generation of the biggest contour, identification of extreme points, cropping of relevant regions, and segmenting the objects using adaptive thresholding.

**Results and Discussion:**

During experimentation, it is found that the EfficientNetB3 model outperforms in accuracy, loss, as well as root mean square error by obtaining 99.96%, 0.149, and 0.386 respectively. However, other models also show the good performance in terms of precision, recall, and F1 score, with high scores close to 0.98 or 1.00, except for VGG19. The findings of the paper emphasize the prospective of deep transfer learning as a viable technique for cotton plant disease diagnosis by providing a cost-effective and efficient solution for crop disease monitoring and management. This strategy can also help to improve agricultural practices by ensuring sustainable cotton farming and increased crop output.

## 1 Introduction

**Figure 1:**
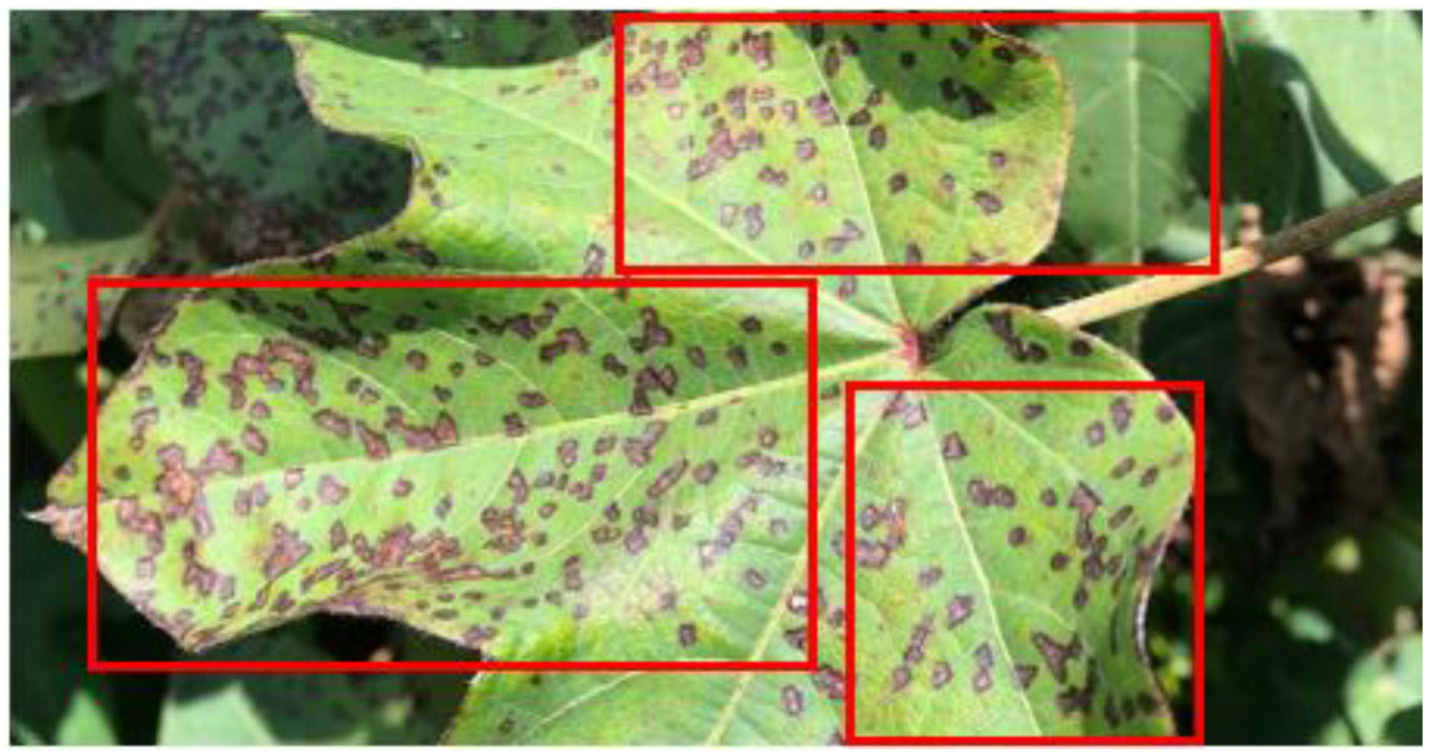
Cotton plant leaf damaged by bacterial blight. The red bounding box highlights the area affected by bacterial blight on the leaf.

Cotton plants are significantly impacted by pests, bacteria or fungal infections which leads to reduced yields and contributes to economic challenges, particularly within the agricultural sector. These diseases pose a serious threat, adversely affecting the development, growth, and overall health of cotton plants. Aphids, Army Worm, Bacterial Blight, Powdery Mildew, and Target Spot are among the most common diseases affecting cotton plants. Aphids are caused by sap-sucking insects that weaken the plant by draining essential nutrients. Army Worm, a pest caused by larvae of *Spodoptera* moths, results in severe leaf defoliation and damage to buds. Bacterial Blight, triggered by *Xanthomonas axonopodis pv. malvacearum*, leads to leaf lesions and reduced fiber quality. Powdery Mildew, caused by fungi like *Erysiphe*, appears as white powdery patches on leaves, hindering photosynthesis. Target Spot, caused by *Corynespora cassiicola*, creates brown circular lesions, reducing yield. Each disease significantly impacts crop health and productivity (Arthy et al., 2024) (**Figure 1** *shows the leaf of cotton plant being damaged by bacterial blight*). These diseases cause significant damage to the crops and decrease its fibre quality as well as throws farmers who deal with the cotton plants in a financial loss. Hence, it is very important to timely, efficiently, and precisely identify such diseased cotton plants (Bhatti et al., 2020). Various traditional techniques are available such as laboratory testing, visually inspecting, etc but they have certain limitations also. Examining any disease on the cotton plant visually is mostly prone to errors as it is completely based on the skill or expertise of a human being (Caldeira et al., 2021).

In fact, to differentiate symptoms caused by these infections from those caused by other factors, such as dietary deficits or environmental stress, can be also difficult. Likewise, laboratory testing is more objective, thereby it is time-consuming and expensive. Moreover, collecting, transporting, and processing under specialized facilities samples can also cause delays in diagnosis and disease management (Zhu et al., 2022).

In these years, deep learning techniques have proven to be as a viable strategy for classifying image-based disease in a multiple fields like plant pathology. By using neural networks, especially convolutional neural networks (CNNs) (Ferentinos, 2018), deep learning models can automatically pull-out hierarchical information from digital images of diseased cotton plant parts like leaves and stems. This image-based approach can learn discriminative features, handle large datasets, and generalize well to previously unseen data. We may accomplish precise and efficient classification of cotton plant diseases using deep learning models trained on labeled datasets of cotton plant pictures, providing farmers, agronomists, and researchers with timely and trustworthy information for disease management methods (Bera and Kumar, 2023; Montalbo, 2022).

Numerous research endeavors have focused on leveraging deep learning methodologies to address the challenge of identifying and classifying diseases affecting cotton plants. Kumar et al. (2022) integrated Convolutional Neural Networks (CNNs) into a mobile application designed to assist farmers to identify and recommend cotton diseases and suitable pesticides respectively. They achieved this by converting the TensorFlow Tflite model (Google AI Edge, 2024) into a Core ML model (Apple Developer, 2024) for seamless integration with iOS apps. Similarly, Memon et al. (2022) proposed a meta-Deep Learning model using a dataset of 2,385 images of both healthy and diseased cotton leaves. They expanded the dataset to enhance the performance of model which resulted in a notable accuracy rate of 98.53%, surpassing other models tested on the Cotton Dataset. Rajasekar et al. (2021) addressed the challenge of detecting cotton plant diseases by developing a hybrid network combining ResNet and Xception models. Their approach outperformed existing techniques, with ResNet-50 achieving a training accuracy (0.95) and a validation accuracy (0.98), along with a training loss (0.33) and a validation loss (0.5). In another study, Naeem et al. (2023) detected, identified, as well as diagnosed cotton leaf diseases using the Root Mean Square Propagation (RMSprop) as well as Adaptive Moment Estimation (ADAM) optimizers. Apart from this, they combined Inception and VGG-16 as feature extractors which resulted in the highest mean accuracy, with the CNN achieving an overall accuracy of 98%. Kalaiselvi and Narmatha (2023) worked on 400 images of cotton plant disease and segmented them using fuzzy rough C-means (FRCM) clustering technique combined with CNN for classification. The researchers demonstrated a remarkable 99% accuracy in diagnosing diseases like Bacterial Blight and Cercospora Leaf Spot. Meanwhile, (Odukoya et al. (2023)) utilized image processing techniques, including watershed segmentation, Edge Detection, Support Vector Machine as well as K-Means Clustering, to detect fungal infections in cotton plants. Arathi and Dulhare (2023) leveraged the DenseNet-121 pre-trained model for enhanced disease classification in cotton leaves, achieving a classification accuracy of 91%. Gülmez (2023) focused on analyzing leaf images to determine plant health, employing the Grey Wolf Optimization technique to identify the most efficient model architecture. Hyder and Talpur (2024) reported a high accuracy rate of 95% using deep transfer learning techniques on a dataset of over 10,000 images of cotton plants. Their model’s effectiveness stemmed from its training on a large-scale dataset. Kukadiya et al. (2024) used a hybrid model combining VGG16 + InceptionV3 to early detect diseases in cotton leaf. By optimizing hyperparameters such as number of epochs and learning rate using stochastic gradient descent (SGD), their ensemble model achieved superior performance with 98% as training accuracy and 95% testing accuracy. Similarly, Mohmmad et al. (2024) worked on the identification of five different cotton crop diseases which includes Aphids, Bacterial Blight, Curly Leaves, Powdery Mildew, and Verticillium Wilt—along with a healthy class. They had dataset of 1,200 images of cotton leaves and were applied on VGG-16, MobileNet, VGG-19, and custom CNN models for classification. These efforts underscore the potential of deep learning in accurately identifying and classifying diseases in cotton leaves, offering effective solutions for agricultural management.

Although the models have demonstrated strong performance, they also face several limitations. One major issue is the use of relatively small or homogenous datasets, which can constrain the generalization and robustness of the models. This limitation is particularly evident when some models achieve high accuracy while others exhibit lower accuracy, as they may be prone to overfitting due to insufficient data diversity. Furthermore, despite some models showing promising results, there is a notable lack of comprehensive comparative analysis between different meta-architectures and optimization strategies. Addressing these gaps could provide valuable insights into model performance and guide the development of more robust and generalizable solutions (Memon et al., 2022; Gülmez, 2023; Naeem et al., 2023; Hyder and Talpur, 2024; Mohmmad et al., 2024).

Hence, the motivation for using proposed models stems from cotton’s pivotal role in global agriculture and the economy. Diseases such as Aphids, Target Spot (a fungal disease), Army Worm, Bacterial Blight, and Powdery Mildew can cause substantial crop losses if not detected early. An automated deep learning-based system offers a more efficient, accurate, and scalable approach to early disease detection. This not only helps safeguard crop yields but also reduces economic losses and promotes sustainability in cotton farming. Apart from this, our work will also try to work on the challenges faced by the existing researchers by conducting comparisons which could provide valuable insights into the most effective approaches for cotton disease detection. Additionally, datasets will also be expanded to encompass a broader range of disease stages, cotton varieties and regularization techniques etc will be used to enhance the execution of model.

In this paper, an automated approach was built that can predict and categorize five types of plant diseases such as Army Worm, Aphids, Bacterial Blight, Target Spot, and Powdery Mildew by evaluating cotton plants alongside healthy ones.

This research has made significant contributions toward achieving this objective:

Comprehensive Image Preprocessing Pipeline: One of the limitations of many existing studies is the use of small or homogeneous datasets, which can hinder model generalization. The paper introduces a robust preprocessing pipeline for handling the COTTON PLANT DISEASE (CPD) dataset, which includes 36,000 images (Dhamodharan, 2023). The preprocessing steps involve removing noise and converting images to grayscale, which enhances the clarity and quality of the data for subsequent analysis. It graphically presents the pre-processed data to reveal patterns and characteristics within the dataset, aiding in a more informed extraction of regions of interest.

Advanced Region Extraction Techniques: The challenge is highlighted regarding the susceptibility of models to overfitting, particularly when there is limited diversity in the data. The paper develops techniques for extracting significant regions of interest from the images by generating the largest contour and identifying extreme points, ensuring that key features are accurately captured. These techniques are employed to further refine the regions of interest, enhancing the precision of the data used for model training and evaluation. Besides this, it will also minimize the risk of overfitting, as the models are trained on well-defined and precise data.

Application and Evaluation of Deep Transfer Learning Models: The study applies a diverse set of advanced transfer learning models - InceptionV3, EfficientNetB3, Xception, ResNet152V2, VGG19, DenseNet169, MobileNetV2, ResNet50V2, EfficientNetB0, as well as InceptionResNetV2, to classify images of healthy leaves and various diseases such as Aphids, Target Spot, Army Worm, Bacterial Blight, and Powdery Mildew. The performance of these models is rigorously evaluated using multiple metrics including F1 score, accuracy, loss, recall, and precision, all computed through the confusion matrix values. This comprehensive evaluation allows for a detailed comparison of model performance.

Selection of Optimal Model: Based on the evaluation metrics, this work identifies and selects the most effective model to classify cotton plant diseases. This selection is guided by an analysis of the models’ performance metrics, ensuring that the chosen model delivers the highest accuracy and reliability. Moreover, the performance of the optimal model has also been compared based on multiple attributes such as dataset, classes, techniques, and accuracy of the existing work which were not reported in some of the previous papers.

The structure of the manuscript is as followed: Section 1 has been already mentioned as an introduction part, which gives us information about the contribution of the researcher’s in the area of detecting cotton plant disease, Section 2 informs us about the methodology that has been used to develop the model for cotton plant disease detection and classification followed by Section 3 where results are analyzed and discussed. Further, the real time implications, improvements as well as the future scope of this research are presented in Section 4 while as the whole paper is at the end summarized and concluded in Section 5.

## 2 Research methodology

**Figure 2:**
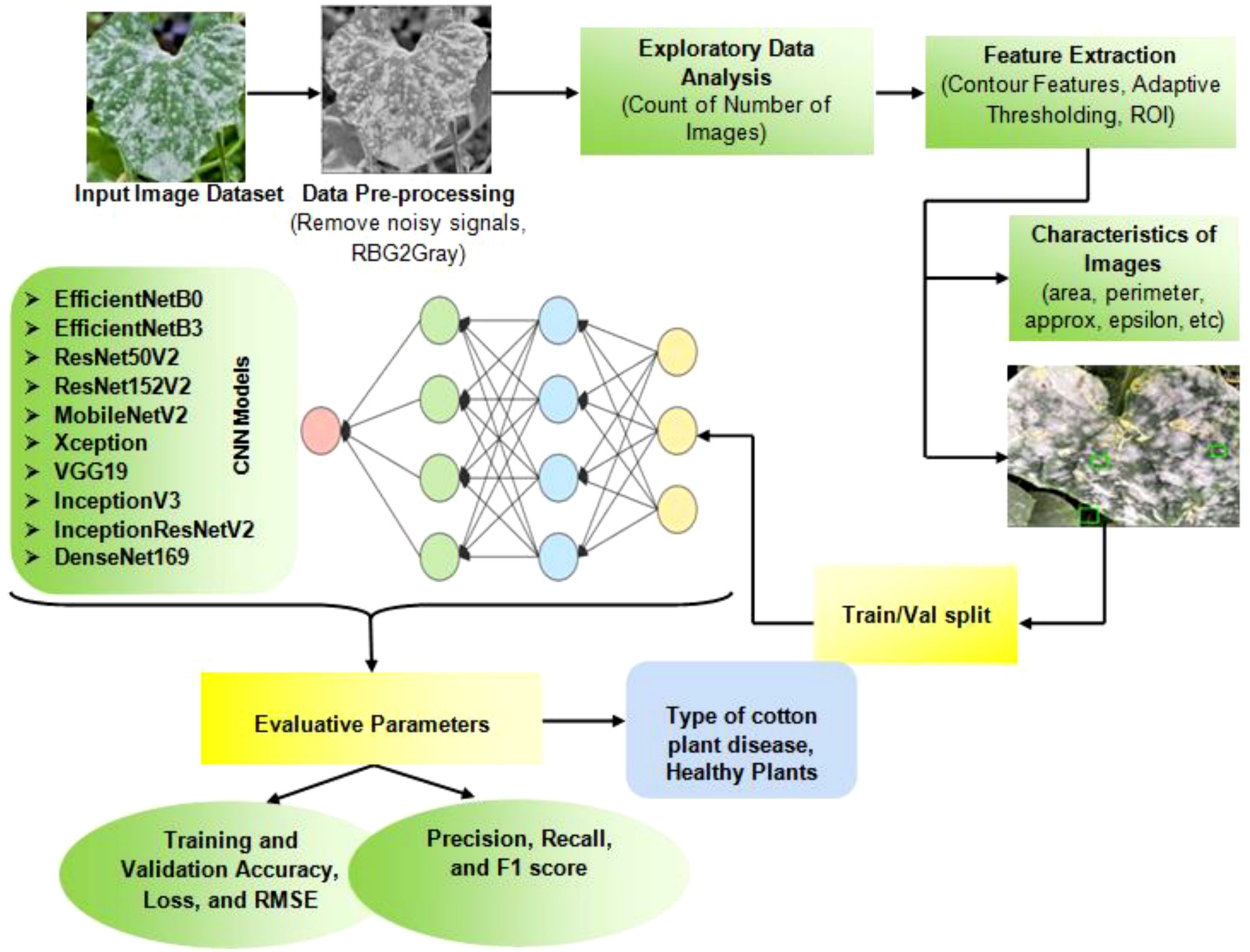
Proposed system to detect cotton plant diseases.

This section focuses on the process of developing a model to identify and classify diseases in cotton plants. The model uses images of the plants and applies several image processing techniques, as well as deep learning classifiers. Apart from this, the novelty of this work lies in the implementation of advanced preprocessing techniques for the Cotton Plant Disease (CPD) dataset. These techniques include comprehensive noise removal, conversion of images to grayscale, and the graphical representation of data to discern underlying patterns. Key preprocessing steps involve extracting Regions of Interest (ROIs) by calculating various image characteristics, identifying the largest contour, locating extreme points, and applying cropping and adaptive thresholding. These refined ROIs are subsequently used as inputs for the deep learning models, thereby enhancing the ability of the model to detect as well as classify plant diseases with improved accuracy. **Figure 2** displays the structure of the proposed model.

A pseudo code (**Algorithm 1**) in disease detection in cotton plants using deep learning techniques has also been mentioned. It outlines the flow of steps involved in training various advanced CNN models for classifying images of healthy and diseased cotton leaves. Let *D* be the CPD dataset containing N=36000  images of healthy leaves and diseased leaves labeled as Aphids, Target Spot, Army Worm, Bacterial Blight, Powdery Mildew, and healthy leaves of cotton plant.

Algorithm 1 **Algorithmic flow of cotton plant disease detection and classification using Deep Transfer Learning Models.**

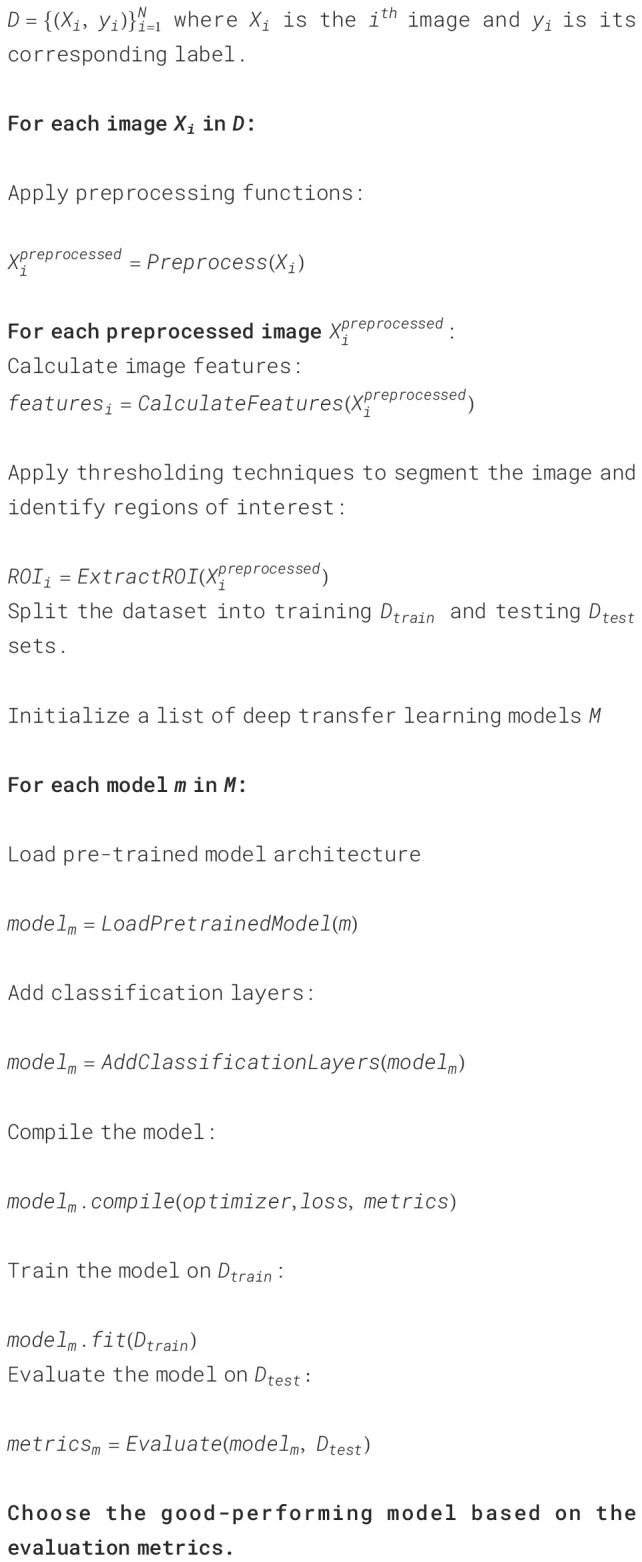

In summary, the proposed system demonstrates a well-structured approach to use advanced image preprocessing techniques and deep transfer learning models for the detection of cotton plant diseases. The application of these methods ensures improved accuracy, minimizing the risks of overfitting and maximizing model performance. Thus, the subsequent section will detail utilized in this study, highlighting its significance in the context of cotton plant disease detection.

### 2.1 Dataset

**Figure 3:**
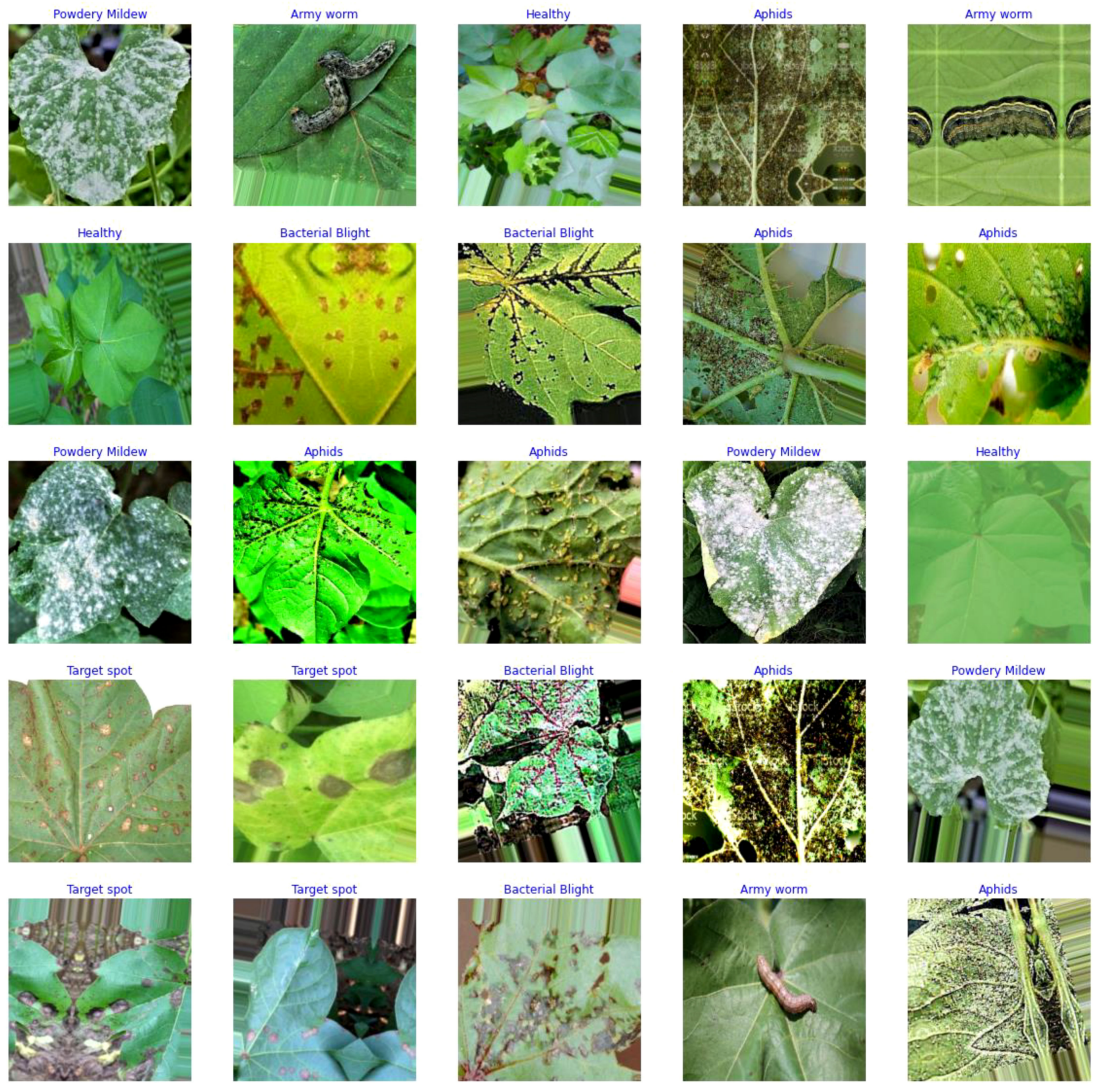
Samples of cotton plant disease image dataset.

The data for the detection and classification of diseases in cotton leaves have been taken from the Cotton Plant Disease database (Dhamodharan, 2023). It is a dataset hosted on the Kaggle platform, which is a popular online community for data scientists and machine learning practitioners. The dataset focuses on diseases affecting cotton plants which include Army Worm, Aphids, Target spot, Powdery Mildew, and Bacterial Blight. It also includes healthy leaf dataset for comparison with the diseased plant. **Figure 3** represents some samples of disease affected cotton leaves which have been taken from the dataset.

The dataset mainly focuses on the disease which occurs only on leaves, and it does not have any reference images for diseases on stem, buds, flowers and boll. It is intended to aid researchers, scientists, and agricultural experts in studying and understanding different diseases that commonly affect cotton crops. By providing access to this dataset, the aim is to enable the development of machine learning models and data-driven approaches to help diagnose and manage cotton plant diseases effectively.

### 2.2 Data pre-processing

**Figure 4:**
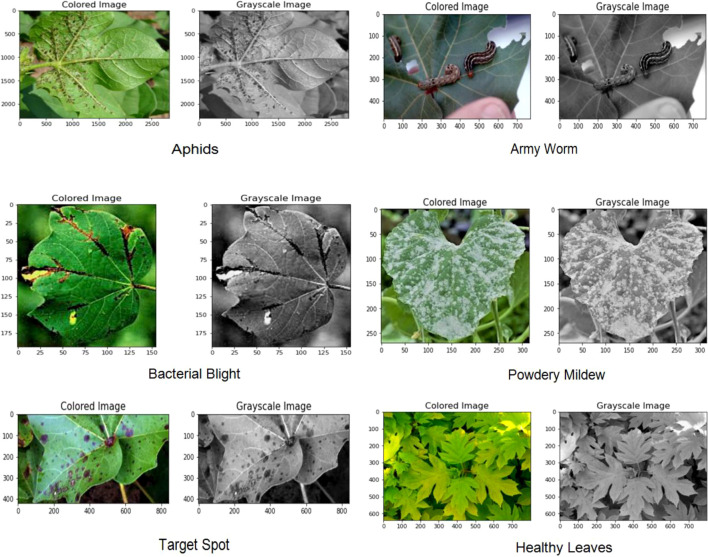
Pre-processing of cotton plant images.

In computer vision and image analysis tasks, pre-processing of cotton leaves images is a critical step for improving the quality of the images and facilitate subsequent analyses. OpenCV, a widely used library in computer vision, is employed due to its rich collection of functions and tools tailored for such tasks (Kotian et al., 2022). In the preprocessing phase for cotton leaf image analysis, the images are first converted to grayscale to simplify processing and reduce computational complexity (**Figure 4**). The grayscale conversion is achieved using the following mathematical formula:


(1) $$I_{gray}=0.299.I_{R}+0.587.I_{G}+0.114.I_{B}$$

where $I_{R}$ , $I_{G}$ , and $I_{B}$ represent the intensity values of the red, green, and blue channels of a pixel, respectively. The coefficients (0.299, 0.587, and 0.114) are derived from the luminance contribution of each color channel in the human visual system. This formula converts the RGB image into a single-channel grayscale image, effectively reducing its dimensionality while retaining the essential visual information necessary for further analysis.

Following the grayscale conversion, Gaussian blurring is applied using a Gaussian kernel to create a smoothed version of the image. This step reduces high-frequency noise and artifacts, enhancing the overall clarity of the image and making it easier to identify relevant features during subsequent analyses. The Gaussian blurring operation is mathematically represented by the convolution of the image with a Gaussian kernel:


(2) $$I_{blurred}\;\left(x,y\right)=\sum_{i=-k}^{k}\sum_{j=-k}^{k}\;I\left(x+i,y+j\right).G\left(i,j\right)$$

where G(i,j) represents the Gaussian kernel function centered at (i,j) and *k* is the kernel radius. This convolution operation smooths the image, aiding in noise reduction and improving feature detection.

The combination of grayscale conversion and Gaussian blurring contributes to the overall goal of preparing the images for analysing it further and classifying the tasks in a computationally efficient manner.

### 2.3 Feature extraction

After examining the dataset and its quality, the next main step is to extract region of interest from each image so that the model can be trained well in future. In this paper, Contour feature extraction has been used which is a fundamental technique in image processing and computer vision used to capture essential shape and boundary information of objects within an image. Contour features are characteristics derived from the contours of regions of interest or object within an image. Contours represent the boundaries of connected components with the same intensity or color. By extracting contour features, we can quantitatively describe the shape and characteristics of these objects, providing valuable information for various image analysis tasks.

In this paper, we have calculated various parameters of an image such as width, area, aspect ratio, height, maximum and minimum value location, mean intensity, extreme right and leftmost, bottom and topmost values etc. in **Table 1**. The area is calculated from the height and width of the object’s bounding rectangle, with height and width derived from the bounding rectangle function. The perimeter measures the distance around the object, and epsilon represents an approximation accuracy for contour approximation. Aspect ratio is the proportion between the width and height of the object, while extent is the ratio of the object’s area to the area of its bounding rectangle. The equivalent diameter corresponds to the diameter of a circle with the same area as the object. Minimum and maximum values, along with their locations, are obtained using specific OpenCV functions, and the mean color of the object is computed from pixel values. The extreme leftmost point is the farthest left coordinate of the contour. These parameters help in detailed shape analysis and characterization of objects in images. The extreme rightmost point of an object is the contour point with the maximum x-coordinate value, representing the farthest point to the right. Similarly, the extreme topmost point is the contour point with the minimum y-coordinate value, which is the highest point on the object. The extreme bottommost point is the contour point with the maximum y-coordinate, marking the lowest part of the object. These points are useful for understanding the spatial boundaries of an object in image analysis and can be obtained using contour analysis functions in OpenCV.

**Table 1:** Characteristics of cotton plant disease image dataset.

Parameters	Formulae	Aphids	Powdery Mildew	Healthy	Target Spot	Army Worm	Bacterial Blight
***Area***	height∗width	1.0	4.0	0.5	2.0	1.0	343.5
***Height***	cv2.boundingRect(cnt)	1	2	2	3	1	20
***Width***	cv2.boundingRect(cnt)	1	5	2	3	1	31
***Perimeter***	$\sqrt{({\left(x_{2}-x_{1}\right)}^{2}+{\left(y_{2}-y_{1}\;\right)}^{2}}$	0.0	10.0	3.41	6.82	0.0	87.35
***Epsilon***	0.1∗cv2∗arclength(cnt, True)	0.0	1.0	0.34	0.68	0.0	8.73
***Aspect ratio***	$\frac{width}{height}$	1.0	2.5	1	1.0	1.0	1.55
***Extent***	$\frac{objectarea}{boundingrectanglearea}$	0.0	0.4	0.12	0.22	0.0	0.55
***Equivalent diameter***	$\sqrt{\frac{4*contourarea}{\pi }}$	0.0	2.25	0.79	1.59	0.0	20.91
***Minimum Value***	cv2.min()	129.0	128.0	128.0	129.0	129.0	128.0
***Maximum Value***	cv2.max()	129.0	132.0	139.0	138.0	129.0	168.0
***Minimum value location***	cv2.minMaxLo()	(2081, 2301)	(127, 269)	(666, 663)	(312, 418)	(327, 480)	(130, 179)
***Maximum Value Location***	cv2.MaxminLo()	(2081, 2301)	(124, 269)	(667, 662)	(311, 418)	(327, 480)	(122, 183)
***Mean color***	cv2.mean()	129.0	129.8	132.0	133.0	129.0	147.90
***Extreme leftmost point***	tuple(cnt(cnt[:, :, 0].argmin()[0])	(2081, 2301)	(123, 268)	(666, 662)	(311, 417)	(327, 480)	(111, 193)
***Extreme rightmost point***	tuple(cnt(cnt[:, :, 0].argmax()[0])	(2081, 2301)	(127, 269)	(667, 662)	(313, 419)	(327, 480)	(141, 191)
***Extreme topmost point***	tuple(cnt(cnt[:, :, 1].argmin()[0])	(2081, 2301)	(123, 268)	(666, 662)	(311, 417)	(327, 480)	(121, 174)
***Extreme bottommost***	tuple(cnt(cnt[:, :, 1].argmax()[0])	(2081, 2301)	(123, 269)	(666, 663)	(311, 419)	(327, 480)	(111, 193)

The parameters shown include key geometric and color properties calculated for each contour detected in the image classes (Aphids, Powdery Mildew, Healthy, Target Spot, Army Worm, and Bacterial Blight). These values aid in object classification by capturing contour dimensions (area, height, width, perimeter, etc.) and color characteristics (mean, min/max values), contributing to the feature extraction used for model training and validation. Bold values present the best values computed by the model.

**Figure 5:**
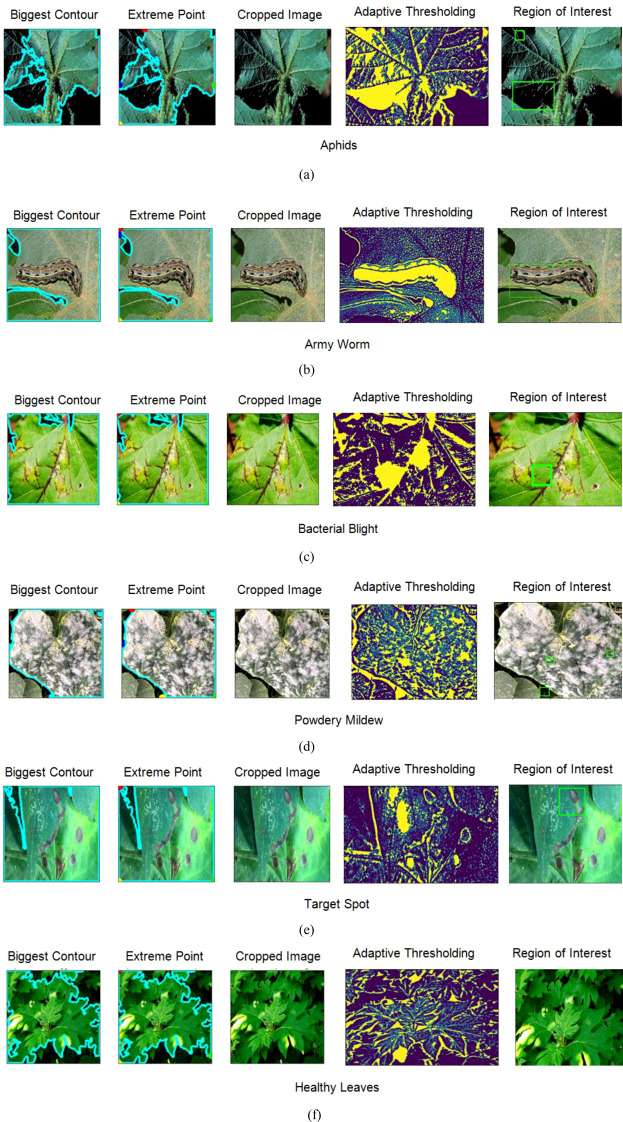
Obtaining region of interest from the images using feature extraction techniques **(A)** Aphids **(B)** Army Worm **(C)** Bacterial Blight **(D)** Powdery Mildew **(E)** Target Spot **(F)** Healthy Leaves.

After computing the characteristics of an image using contour features, the next steps involve several image processing techniques for further analysis and extraction of regions of interest (ROIs) as shown in **Figure 5**.

First, the biggest contour, representing the most prominent object in the image, is identified. This can be achieved by finding the contour with the largest area among all detected contours. In the second step, the extreme points of this biggest contour are generated in the form of a continuous curve around the object. Later, the images are cropped within the boundary of those curves to isolate it from the rest of the features.

In the next phase, adaptive thresholding is applied to the cropped image to adjust the threshold value for each pixel in the image based on its local neighbourhood to enhance the quality of an image. In addition to this, it also improves the segmentation of the object from the background, most particularly in those cases where there is a variation in the lighting conditions or uneven illumination. Finally, the last step is obtaining the region of interest for which another contour detection technique is applied.

This contour delineates the boundary of the main object, which can be further utilized for shape analysis, feature extraction, or object recognition tasks. The combination of all these techniques provides a systematic approach to extract and analyse specific regions in the form of features from the original image, for providing more accurate image analysis and computer vision tasks. Later the dataset has been split into training and validation in 80:20 based on which the performance of the applied models has been examined.

### 2.4 Applied deep learning classifiers

This section provides a concise overview of the many deep learning classifiers that have been used for the detection and classification of cotton plant diseases. Additionally, the section also defines the hyperparameters used to build model during training it with the cotton plant disease dataset, as presented in **Table 2**.

**Table 2:** Hyperparameters used in deep learning models.

Hyperparameter	Value
Learning_Rate	0.001
Batch_size	32
Number of Epochs	10
Optimize	Adam
Dropout	0.5
Activation Function	ReLU and Sigmoid
Kernel Size	3x3
Loss	Categorical_crossentropy
Image_size	224 x 224

EfficientNet is a family of CNNs that significantly improve the efficiency of deep learning models through a combination of model scaling, depthwise separable convolutions, and a compound scaling method (Tan, 2019). EfficientNet employs a novel approach to balance network depth, width, and resolution, to achieve performance on various benchmarks during the maintenance of computational efficiency. The key innovation of EfficientNet lies in its compound scaling method, which uniformly scales all dimensions of the network, leading to a better trade-off between accuracy and computational cost. This architecture has been particularly effective in a range of computer vision tasks due to its ability to deliver high performance with less parameter compared to traditional deep learning models.

In paper two versions of EfficientNet model i.e. EfficientNetB0 and EfficientNetB3 have been used (Babu and Jeyakrishnan, 2022; Sun et al., 2022). EfficientNetB0 is a convolutional neural network architecture made up of a stem convolution and a base architecture with repeating building blocks that allows it to successfully extract features in a hierarchical order. Custom dense layers are added to adapt it for cotton plant disease detection, followed by an output layer with a softmax activation function to match the number of classes. Transfer learning allows the use of already trained weights from ImageNet as well as data augmentation techniques and appropriate optimisation approaches to improve the model’s performance during training. By leveraging EfficientNetB0 and fine-tuning hyperparameters, one can achieve robust and accurate cotton plant disease detection and classification (Sun et al., 2022). On the contrary, EfficientNetB3 is a type of CNN that provides promising results to detect as well as classify cotton plant diseases. The architecture has a balancing width, depth, resolution, and compound scaling approach, for optimizing the performance. This model comprises of multiple blocks of depthwise separable convolutions, which split channel-wise and spatial convolutions for reducing the computational complexity. These blocks enable the model to learn hierarchical features at different scales, crucial for identifying disease-related patterns in cotton plant images. For multi-class classification, the architecture includes a global average pooling layer and fully connected layers, along with softmax activation. The model focuses on achieving accurate and efficient detection along with the classification of multiple diseases in cotton plant images in order to empower agricultural applications for identifying diseases at their earliest stages and improving crop management.

DenseNet is a convolutional neural network architecture characterized by its dense connectivity pattern (He et al., 2016). Unlike traditional networks where each layer receives input from the previous layer, DenseNet works in a feed forward fashion by connecting each layer to every other layer. This dense connectivity improves gradient flow, reduces the risk of vanishing gradients, and encourages feature reuse, leading to more efficient training and better performance. DenseNet models are known for their reduced number of parameters compared to other deep networks while maintaining high accuracy, making them effective for a variety of image recognition tasks. In this research, its variant has been used which is DenseNet169 (Akbar et al., 2023; Huang et al., 2017). It presents an effective solution for detecting and classifying cotton plant diseases. The architecture is characterized by promoting strong feature reuse, densely connected layers, and gradient flow across the network, leading to better parameter efficiency and performance. A comprehensive dataset comprising of images of both healthy and diseased cotton plants is gathered and categorized with corresponding disease classifications, specifically for the purpose of detecting and classifying cotton plant diseases. DenseNet-169’s distinctive dense blocks, each with 32 growth rate feature maps, facilitate the extraction of intricate and disease-specific features from the input images. Between dense blocks, transition blocks with convolutional layers and average pooling help manage computational complexity while maintaining relevant information flow.

Xception, short for “Extreme Inception,” is designed to enhance existing convolutional networks by employing depthwise separable convolutions (Rai and Pahuja, 2024). This architecture effectively combines spatial and channel-wise filtering, resulting in improved computational efficiency and performance. The model is based on the idea of Inception modules, but instead of using regular convolutions, it employs depthwise separable convolutions. This technique significantly decreases the number of parameters as well as computational complexity. Xception is made up of several depthwise separable convolution blocks, each containing residual connections for improved gradient flow and information propagation.

MobileNetV2 has an architecture that is lightweight and built for efficient and quick performance on mobile and embedded devices (Verma and Singh, 2022). The usage of inverted residual blocks, which are designed to balance computational efficiency and model correctness, is a significant component of MobileNetV2. These blocks use stride in the depthwise separable convolution followed by a pointwise convolution with expansion and squeeze-and-excitement operations to improve how features are represented and how information flows. MobileNetV2 also uses linear bottlenecks to reduce computing costs while preserving performance. The architecture is designed to strike a balance between model size, speed, as well as accuracy, which makes it well-suited for the applications and environments in real time with limited number of resources.

ResNet (Residual Networks), revolutionized deep learning with its introduction of residual blocks, which help mitigate the vanishing gradient problem in very deep networks (He et al., 2016). ResNet allows for the training of extremely deep networks by introducing shortcut connections that skip one or more layers. These residual connections enable gradients to flow more effectively through the network during backpropagation, which significantly improves training efficiency and model performance. ResNet architectures are known for their remarkable ability to achieve high accuracy on various image recognition tasks and have set new benchmarks in computer vision competitions, making them a fundamental building block in modern deep learning.

The extended version of ResNet is ResNet50V2V2 and ResNet152V2 (Ahad et al., 2023; Kumar et al., 2023). The architecture of ResNet50V2 consists of 50 layers, comprising multiple stacked residual blocks with increasing complexity. The first few layers could perform initial feature extraction from input images of healthy and diseased cotton plants. The subsequent layers would progressively learn more abstract and intricate disease-related features. The network’s final layers would include dense layers for classification, with softmax activation to produce the probabilities for different disease classes. Similarly, another extended version of ResNet152V2. It is an extension of the ResNet architecture with 152 layers, showcasing deeper and more powerful representations. The architecture begins with initial convolutional layers to extract low-level features from input images of healthy and diseased cotton plants. Then, a series of residual blocks with increasing complexity would be stacked on top of each other, enabling the network to learn hierarchical and abstract disease-related features. The residual connections within each block facilitate the smooth flow of information and alleviate the vanishing gradient problem which allows for effective training of very deep networks. Towards the end of the network, global average pooling would be employed to reduce spatial dimensions, and dense layers with softmax activation would perform the final classification into different disease classes.

VGG models are characterized by their simplicity and uniformity (Simonyan and Zisserman, 2014). The VGG network design emphasizes the use of small 3x3 convolutional filters and 2x2 max-pooling layers, which contribute to a deep architecture while keeping the model design straightforward. VGG networks, particularly VGG16 and VGG19, have become widely used due to their high performance in classifying images and their ability to serve as powerful feature extractors for various transfer learning applications. Their straightforward architecture and high accuracy make them a popular choice in the deep learning community.

In this paper, VGG19 is being used (Peyal et al., 2022). The architecture has a deep convolutional neural network that can be adapted for the detection and classification of cotton plant diseases. VGG19 consists of 19 layers, including multiple convolutional and max-pooling layers. The architecture follows a simple and uniform design, where each layer contains 3x3 convolutional filters, followed by ReLU activations to introduce non-linearity. Max-pooling layers are used for downsampling and reducing spatial dimensions. Towards the end of the network, fully connected layers are employed for classification, with softmax activation to predict the probabilities of different disease classes. To adapt VGG19 for cotton plant disease detection, the final dense layers would be modified to match the number of disease classes. By training the model on a diverse dataset of cotton plant images, VGG19 would aim to accurately detect and classify various cotton plant diseases, aiding in early disease identification and effective crop management in agriculture.

Inception networks employ the “Inception module” for feature extraction which considers features at different scales and comprise several convolutional filters of different sizes in the same layer. It also enables model to obtain multiple forms of representation of feature, which is advantageous. Inception networks are incredibly popular due to their high effective and computational ability in image classification as well as other computer vision application. InceptionV3 model is a deep convolutional neural network and it has been found to be useful for the detection and classification of diseases affecting cotton plant (Mary et al., 2022). It is an improvement of the others previously created by Google Research such as Inception and InceptionV2 and aims at achieving a good level of accuracy in addition to ensuring efficient use of computations through its implementation. There are several inception modules, which are convolutional blocks with multiple branches of different kernel size to make it able to capture features of different scales. The same concept exists in InceptionV3 where factorization has been performed to split them into more compact convolutions. For detecting the cotton plant diseases, the last dense layers of InceptionV3 can be trained according to the specific diseases class to help in the agricultural applications & improving crop management.

Hybrid (InceptionResNetV2) incorporates concepts from both the Inception and ResNet models (Sadiq et al., 2023). The architecture incorporates residual connections as well as inception modules via ResNet and the inception family, respectively. InceptionResNetV2 is made up of deep convolutional layers followed by inception blocks with several branches and varying kernel sizes for feature extraction. Furthermore, residual blocks are used to ensure that information flows smoothly across the network, to allow for the effective training of very deep models. InceptionResNetV2 is well-known for its excellent accuracy and is extensively used in classification of images, detecting objects, and segmentation-like applications in computer vision. However, due to its complex nature, it may necessitate significant computer resources for computation.

In addition to this, **Table 3** provides a comparison of various applied models by detailing their total number of parameters which includes the breakdown of trainable and non-trainable parameters. This information helps to understand the computational complexity and potential performance of each model during training and validation. Here, VGG19 has the highest number of parameters at 143.67 million, all of which are trainable, indicating a highly complex model but potentially more prone to overfitting. ResNet152V2 follows with over 60 million parameters, emphasizing its depth and strong learning capacity. In contrast, models like MobileNetV2 and EfficientNetB0 have significantly fewer parameters (3.53 million and 5.27 million, respectively), making them more efficient for real-time applications, though possibly sacrificing some accuracy due to reduced complexity. Xception, InceptionV3, and DenseNet169 strike a balance between complexity and performance, with substantial parameter counts but remains computationally feasible.

**Table 3:** Summary of the applied models.

Models	Total Parameters	Trainable Parameters	Non-Trainable Parameters
**EfficientNetB0**	5270571	5228548	42023
**DenseNet169**	14307880	14149480	158400
**Xception**	22910480	22855952	54528
**MobileNetV2**	3538984	3504872	34112
**ResNet50V2**	25613800	25561512	52288
**ResNet152V2**	60419944	60348520	71424
**Vgg19**	143667240	143667240	0
**InceptionV3**	23851784	23819784	32000
**EfficientNetB3**	12320919	12244549	76370
**InceptionResNetV2**	55873736	55823848	49888

Bold values present the best values computed by the model.

### 2.5 Performance metrics

Performance metrics are used to examine the quality and the performance of the models for any data. These metrics provide quantitative measures that assess how well a model performs its intended task, such as classification or regression. The selection of performance measures is based upon the specific scenario and the objectives of the analysis. Here are the metrics that have been used to evaluate the performance of the models (Latif et al., 2021; Elaraby et al., 2022; Kinger et al., 2022; Nalini and Rama, 2022; Kanna et al., 2023):

Accuracy is a broad measure of how well a model performs in correctly identifying positive and negative examples. If the accuracy is having greater value, it suggests that the classification done is very precise while as Loss is a mathematical function that measures the difference between the predicted values of a model as well as the actual values in the training data. Similarly, Root Mean Square Error is defined as the square root of the mean of the squared residuals, which are the differences between the predicted values as well as the true values. Moving to another set of parameters, Precision provides information related to the ability of the model for avoiding false positives. It means that informs the correctly identification of positive instances without generating large number of incorrectly positive predictions. Recall is used for examining the ability of model to identify accurately the positive classes. In short, recall, enables to identify all positive instances and avoids false negatives whereas F1 Score is a performance metric that considers both precision and recall, giving a balanced measure of how accurate a classification model is.

## 3 Results

The section of this research paper serves as the focal point where empirical findings are presented, unveiling the outcomes of rigorous investigations and data analysis. After framing and executing the objectives and methodology of our study in the preceding sections, we now turn our attention to the fundamental outcomes that emerged from our investigation. In this section, we have presented a concise and informative analysis of the applied deep transfer learning models based on different performance measures as mentioned in section (2.5) for both training as well as validation dataset.

Initially we conducted a complete evaluation of various applied deep learning models for the whole cotton plant disease dataset. To calculate the efficacy of each model, we rigorously measured three critical performance metrics: accuracy, loss, and root mean square error across both the training as well as validation datasets. Employing a ten-epoch training regimen, we obtained valuable insights into the learning dynamics and convergence patterns of each model, as shown in **Table 4**. Such detailed assessment enables us to make informed decisions about model selection, parameter tuning, and potential avenues for further improvement.

**Table 4:** Execution of applied techniques for CPD dataset.

Techniques	Training			Validation		
	Accuracy	RMSE	Loss	Accuracy	Loss	RMSE
**EfficientNetB0**	99.81	0.461	0.213	99.16	0.198	0.444
**DenseNet169**	**99.86**	**0.434**	**0.189**	98.88	0.201	0.448
**Xception**	99.70	0.451	0.204	99.16	0.197	0.443
**MobileNet V2**	99.45	0.457	0.209	99.80	0.175	0.418
**ResNet50 V2**	99.76	0.496	0.247	99.87	0.189	0.434
**ResNet152 V2**	98.49	0.563	0.317	97.94	0.298	0.545
**VGG19**	76.97	0.964	0.930	74.13	0.961	0.980
**Inception V3**	99.55	0.473	0.224	99.58	0.211	0.459
**EfficientNetB3**	99.81	0.435	0.190	**99.96**	**0.149**	**0.386**
**Hybrid (InceptionResNet V2)**	99.50	0.533	0.285	99.95	0.221	0.470

Bold values present the best values computed by the model.

Among the models examined during training phase, DenseNet169 demonstrated remarkable accuracy by achieving 99.86% and similarly, EfficientNetB0, EfficientNetB3, MobileNetV2, Xception, ResNet50V2, InceptionV3, and InceptionResNetV2 also exhibited the high accuracy rates of 99.81%, 99.81%, 99.45%, 99.70%, 99.76%, 99.55%, and 99.50% respectively and also surpasses 99% benchmark. Likewise, for validation phase, the top accuracy has been obtained by EfficientNetB3 with 99.96% closely followed by InceptionResNetV2 having accuracy of 99.95%. Similarly, EfficientNetB0, Xception, MobileNetV2, ResNet50V2, and InceptionV3 maintained accuracy levels within the range of 99% to 100%, which signifies their strong generalization capabilities. However, a notable exception was ResNet152V2 and VGG19, which exhibited displayed relatively lower accuracies where their values dropped to 98.49%, 76.97% during the training phase and 97.94%, 74.13% during validation phases, respectively. These results suggest that the layers of the ResNet152V2 and VGG19 model may not have been effectively trained and are struggling to capture essential patterns in both the training as well as validation datasets.

Moving to the loss values, they represent the discrepancy between predicted and actual outputs, indicating how well the models fit the data. Notably, DenseNet169 showcased the lowest loss during both training (0.189) and EfficientNetB3 during validation phase (0.149), which implies a superior fit to the dataset. On the other hand, VGG19 had significantly higher losses, reaching 0.930 during training and 0.961 during validation, indicating poorer model performance in capturing the data patterns.

Furthermore, the RMSE values which assess the average magnitude of errors in our predictions, has been also computed. On assaying, it has been found that EfficientNetB3 demonstrated the lowest RMSE (0.386) during validation, reflecting its ability to make precise predictions on continuous data. In contrast, VGG19 exhibited the highest RMSE values as compared to the other models, both during training (0.964) and validation (0.980), indicating less precise predictions.

**Figure 6:**
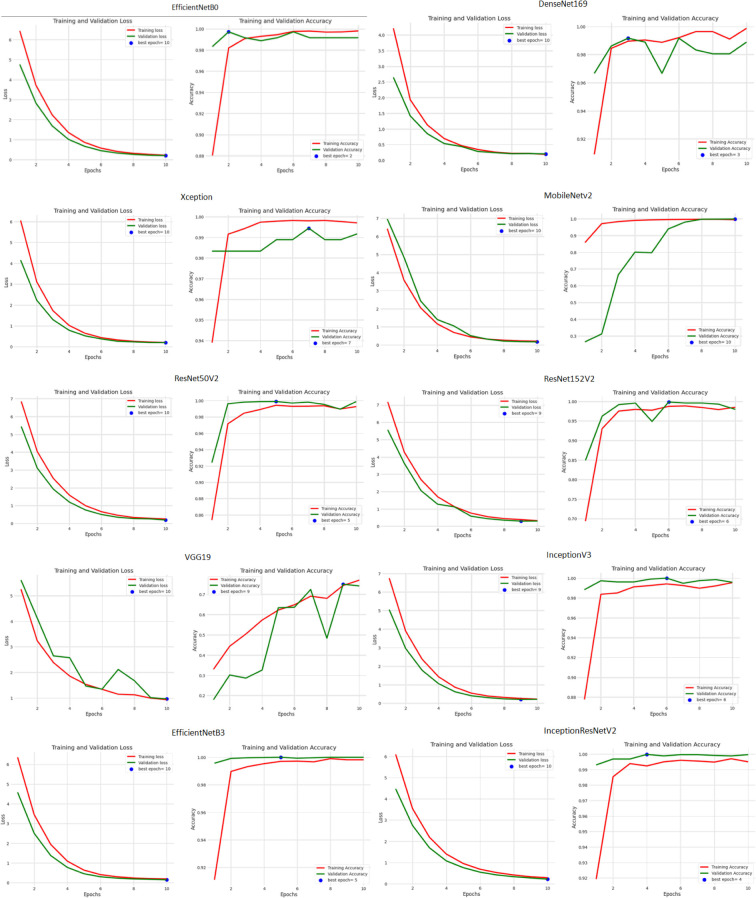
Analysis of techniques based on their graphical curves.

Besides this, the accuracy and loss performance of the models are also examined based on their curves that have been generated during both training as well as validation phases in **Figure 6**.

From the analysis of the training and validation process depicted in the figure, it is evident that epoch 10 yielded the lowest training and validation loss values across all the models, indicating that the models achieved their good performance in terms of minimizing the difference between actual and predicted values at this epoch. This suggests that after 10 epochs of training, the models converged to a state of optimized loss values. However, when examining the accuracy metric, a more diverse pattern emerges. Different models reached their peak accuracy scores at various epochs during the training process. EfficientNetB0 attained its highest accuracy at epoch 2, DenseNet169 at epoch 3, Xception at epoch 7, ResNet50V2 at epoch 5, ResNet152V2 at epoch 6, InceptionV3 at epoch 6, InceptionResNetV2 at epoch 4, VGG19 at epoch 9, MobileNetV2 at epoch 10, and EfficientNetB3 at epoch 5.

After examining the models for the whole dataset, they have been also evaluated for the different classes of the dataset such as Aphids, Healthy Leaves, ArmyWorm, Powdery Mildew, Bacterial Blight, and Target Spot as mentioned in **Table 5** *(The table provides a comprehensive overview of the results, showing that for the Aphids class, the top performance in training was achieved by DenseNet169, while EfficientNetB0 delivered the best result during validation).*

**Table 5:** Evaluation of techniques for different classes of CPD dataset.

Techniques	Class	Training			Validation		
		Accuracy	Loss	RMSE	Accuracy	Loss	RMSE
**EfficientNetB0**	**Aphids**	99.19	0.213	0.461	**99.75**	**0.199**	**0.446**
**ResNet50 V2**		99.16	0.247	0.496	99.16	0.499	0.706
**Xception**		99.46	0.204	0.451	99.59	0.469	0.684
**MobileNet V2**		99.49	0.209	0.457	99.16	0.159	0.398
**VGG19**		76.49	0.930	0.964	74.79	0.489	0.699
**ResNet152 V2**		98.49	0.317	0.563	97.56	0.526	0.725
**DenseNet169**		**99.49**	**0.189**	**0.434**	98.16	0.496	0.704
**Inception V3**		99.46	0.224	0.473	99.13	0.486	0.697
**EfficientNetB3**		99.49	0.190	0.435	99.00	0.599	0.773
**Hybrid(InceptionResNetV2)**		99.16	0.285	0.533	99.59	0.498	0.705
**EfficientNetB0**	**Army Worm**	98.49	0.493	0.702	99.49	**0.149**	**0.386**
**DenseNet169**		97.59	0.499	0.706	98.46	0.246	0.495
**Xception**		98.46	0.224	0.473	99.49	**0.149**	**0.386**
MobileNet V2		98.77	0.469	0.684	99.16	0.119	0.344
ResNet50 V2		98.49	0.497	0.704	99.49	**0.149**	**0.386**
**ResNet152 V2**		98.20	0.597	0.772	97.76	0.246	0.495
VGG19		78.78	0.490	0.700	74.49	0.949	0.974
Inception V3		**98.89**	**0.264**	**0.513**	**99.52**	0.246	0.495
**EfficientNetB3**		98.15	0.620	0.787	99.49	**0.149**	**0.386**
**Hybrid(InceptionResNetV2)**		98.50	0.345	0.587	99.00	0.248	0.497
**EfficientNetB0**	**Bacterial blight**	98.49	0.429	0.654	99.49	0.149	0.386
**DenseNet169**		97.59	0.459	0.677	98.46	0.246	0.495
**Xception**		99.46	0.250	0.498	**99.59**	**0.149**	**0.386**
**MobileNet V2**		99.77	0.446	0.667	99.16	0.119	0.344
**ResNet152 V2**		99.20	0.578	0.760	97.49	0.246	0.495
**ResNet50 V2**		99.49	0.426	0.652	99.46	0.149	0.386
**Inception V3**		**99.89**	**0.249**	**0.498**	99.49	0.246	0.495
**VGG19**		79.78	0.459	0.677	74.56	0.949	0.974
**EfficientNetB3**		99.15	0.682	0.825	**99.59**	**0.139**	**0.386**
**Hybrid(InceptionResNetV2)**		99.50	0.370	0.608	99.00	0.248	0.497
**EfficientNetB0**	**Target Spot**	98.81	0.493	0.702	**99.59**	0.499	0.706
**DenseNet169**		97.86	0.469	0.684	98.46	0.466	0.682
**Xception**		98.70	0.764	0.874	98.16	0.596	0.772
**MobileNet V2**		98.45	0.199	0.446	98.49	0.769	0.876
**ResNet50 V2**		98.76	0.497	0.704	98.46	0.599	0.773
**ResNet152 V2**		98.49	0.497	0.704	96.59	**0.206**	**0.453**
**VGG19**		78.97	0.990	0.994	74.82	0.469	0.684
**Inception V3**		98.55	0.464	0.681	99.42	0.296	0.544
**EfficientNetB3**		**98.81**	0.490	0.700	98.23	0.469	0.684
**Hybrid(InceptionResNetV2)**		98.50	**0.195**	**0.441**	98.49	0.498	0.705
**EfficientNetB0**	**Powdery Mildew**	**98.59**	0.449	0.670	**99.49**	**0.159**	**0.398**
**VGG19**		78.00	0.948	0.973	74.56	0.599	0.773
**Xception**		98.46	0.720	0.848	99.69	0.146	0.382
**MobileNet V2**		98.49	**0.149**	**0.386**	99.16	0.499	0.706
**ResNet50 V2**		98.15	0.448	0.669	99.46	0.489	0.699
**DenseNet169**		97.49	0.446	0.667	98.46	0.216	0.464
**ResNet152 V2**		98.00	0.446	0.667	97.49	0.486	0.697
**EfficientNetB3**		98.46	0.416	0.644	99.59	0.489	0.699
**Inception V3**		98.49	0.476	0.689	99.49	0.466	0.682
**Hybrid(InceptionResNetV2)**		98.49	0.149	0.386	99.00	0.008	0.089
**EfficientNetB0**	**Healthy Leaves**	99.49	0.249	0.498	**99.49**	**0.159**	**0.398**
**DenseNet169**		99.46	0.146	0.382	98.46	0.216	0.464
**Xception**		99.46	0.276	0.525	98.49	0.146	0.382
**MobileNet V2**		99.75	0.219	0.467	98.00	0.499	0.706
**EfficientNetB3**		99.00	0.150	0.387	98.59	0.489	0.699
**VGG19**		76.79	0.955	0.977	74.56	0.599	0.773
**ResNet152 V2**		98.76	0.376	0.613	96.79	0.486	0.697
**Inception V3**		99.16	0.279	0.528	99.49	0.466	0.682
**ResNet50 V2**		99.49	0.246	0.495	98.49	0.489	0.699
**Hybrid(InceptionResNetV2)**		**99.76**	**0.200**	**0.447**	98.00	0.008	0.089

Bold values present the best values computed by the model.

In **Table 5**, for Aphids class, EfficientNetB0, DenseNet169, Xception, EfficientNetB3, and MobileNet V2 achieved high accuracies during training, with values ranging from 99.19% to 99.49%. However, during validation, their accuracies varied, with EfficientNetB0, Xception, MobileNetV2, ResNet50V2, InceptionV3, EfficientNetB3, and InceptionResNetV2 maintaining accuracies above 99%, while DenseNet169 lower accuracy at 98.16%. However, VGG19 exhibited significantly lower accuracy values of 76.49% and 74.79% during training and validation, respectively, indicating challenges in capturing important features associated with aphids in the dataset. Regarding the loss and RMSE metrics, models such as DenseNet169 and MobileNetV2 demonstrated lower values for training as well as validation phase respectively, while as VGG19 and ResNet152 V2 exhibited higher values which suggest improvement in their prediction.

For ArmyWorm class, all the models except DenseNet169 and VGG19 have computed the accuracies above 97% for training phase, while as during validation phase these same models excluding ResNet152V2 have obtained the accuracies above 98%. On analysing, it has been observed that IncpetionV3 model has the highest validation accuracy of 99.52% while as VGG 19 once again has the lowest accuracy value of 74.49%. Similarly for the loss and root mean square error value, Xception model computed the lowest value of 0.224 and 0.473 respectively during validation phase, and on the contrary, MobileNetV2 obtained the good loss and RMSE score for validation dataset with 0.119 and 0.344 respectively.

Similarly, the models have been also computed for the other classes of the cotton plant disease dataset such as Powdery Mildew, Bacterial Blight, Healthy leaves, and Target Spot using the same metrics. On analyzing the performance of models for these classes, it has been observed that in case of Bacterial Blight, InceptionV3; Target Spot,EfficientNetB0 and InceptionResNetV2; Powdery Mildew,EfficientNetB0 and (MobileNetV2 and InceptionResNetV2); and Healthy Leaves, InceptionResNetV2 and DenseNet169 computed the good accuracies, loss, and RMSE score values respectively. On the other hand, during validation phase the highest accuracy, root mean square error, and loss scores have been generated by EfficientNetB3 for Bacterial Blight, EfficientNetB0 and ResNet50V2V2 for Target Spot, Xception and InceptionResNetV2 for Powdery Mildew, and EfficientNetB0 and InceptionResNetV2for Healthy leaves.

**Figure 7:**
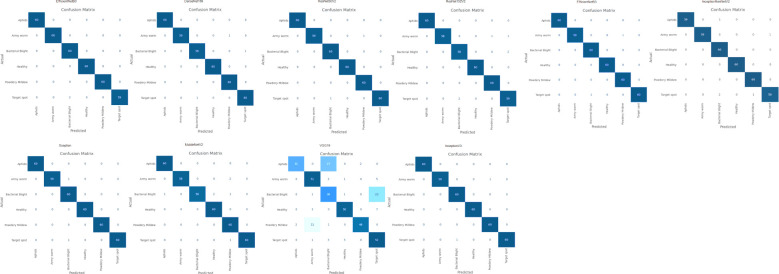
Confusion matrix of applied deep learning techniques.

After examining the models such as EfficientNetB0, EfficientNetB3, ResNet50V2, ResNet152V2, InceptionV3, InceptionResNetV2, VGG19, Xception, MobileNetV2, and DenseNet169 for their accuracies, loss, and RMSE scores during training and validation phase, the goal is to evaluate their performance for different set of metrics i.e. recall, precision, and F1 score. Hence, to assess the effectiveness of these models, **Figure 7** shows a widely used evaluation tool known as the confusion matrix.

We have generated the confusion matrix of 6x6 i.e. 6 rows and 6 columns which signifies the 6 difference classes of cotton disease plants i.e. Aphids, Powdery Mildew, Army Worm, Target spot, Bacterial Blight, as well as Healthy leaves. The matrix contains information about the number of samples that are classified as belonging to class i but are predicted as class j by the model. The diagonal elements (i.e., the elements with i=j) of the confusion matrix represent the true positive values for each class. These values show how many samples have been correctly categorized for each disease class. Simply put, these instances demonstrate when the model accurately identified a sample as belonging to a specific disease class.

Conversely, the off-diagonal elements, where i ≠ j, correspond to the false positive values. These elements signify the number of misclassifications made by the model, where it predicted a sample as a particular disease class when it belonged to a different one. On the contrary, in case of true negative and false negative, they are calculated by summing up all the samples that are correctly classified for all classes other than the specific class being considered and the instances where samples that belong to a specific class are incorrectly predicted as other classes respectively (Wikipedia, 2024).

Hence, analyzing the diagonal sequence of the confusion matrix allows us to measure the model’s accuracy for each disease class individually. Higher values along the diagonal indicate that the model is performing well in correctly identifying samples for those disease classes, while lower values may indicate areas where the model needs improvement. High performance was observed for most models, but certain models, such as VGG19, showed weaker performance with lower precision and recall values.

Based on the values of confusion matrix, other parameters except accuracy and loss of the applied deep transfer learning models have been computed for the complete cotton plant disease dataset as shown in **Table 6**.

**Table 6:** Examination of classifiers for other parameters of performance metrics.

Classifiers	Precision	Recall	F1 score
**EfficientNetB0**	0.99	0.99	0.99
**DenseNet169**	0.99	0.99	0.99
**Xception**	1.00	0.98	0.99
**MobileNet V2**	0.98	0.98	0.98
**Inception V3**	0.99	0.99	0.99
**ResNet152 V2**	0.98	0.98	0.98
**VGG19**	0.78	0.74	0.74
**ResNet50 V2**	0.99	0.99	0.99
**EfficientNetB3**	0.99	0.99	0.99
**Hybrid(InceptionResNet V2)**	0.99	0.99	0.99

Bold values present the best values computed by the model.

Looking at the results, we can observe that most of the models such as EfficientNetB0, DenseNet169, Xception, MobileNetV2, ResNet50 V2, ResNet152 V2, Inception V3, EfficientNetB3, and Hybrid (InceptionResNetV2) have achieved excellent performance, with high precision, recall, and F1 scores close to 0.98 or 1.00. It means that these models have demonstrated outstanding capabilities in accurately classifying images into their respective categories, with minimal misclassifications. However, VGG19 seems to lag the other models, with noticeably lower precision, recall, and F1 scores of around 0.78 and 0.74 respectively. This suggests that VGG19 might struggle with certain classes and is relatively less accurate in its predictions on comparing to the other models.

**Figure 8:**
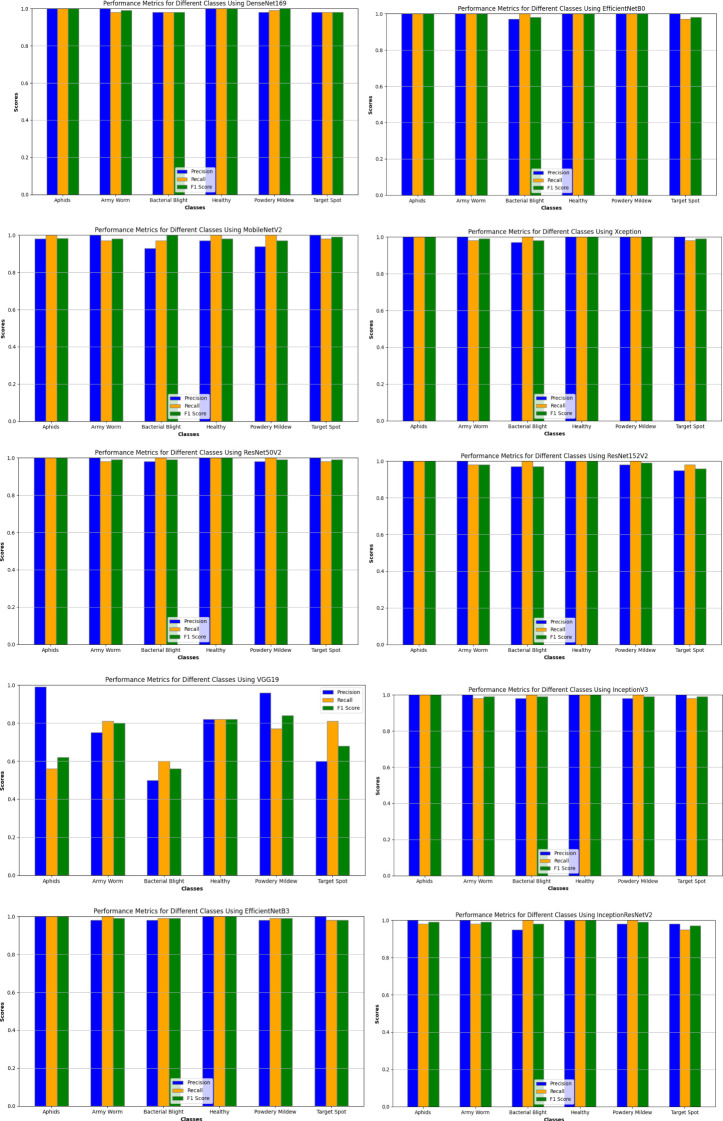
Evaluating performance of deep learning models on various classes of datasets.

Likewise, the execution of the models has been also assayed for the different classes of the dataset based on the same performance metrics as discussed earlier and their results are graphically represented in **Figure 8**.

Upon analyzing the pattern of the graph in the figure, a clear trend emerges, indicating that all the models have generally demonstrated excellent precision, recall, and F1 scores for the six different classes of the dataset, with values ranging between 0.92 to 1.00, except for the VGG19 model. The performance of the VGG19 model varies across different classes, particularly for classes with values below 0.90. Albeit, for the Aphids class, the model exhibits a high precision of 0.94 but struggles with recall at 0.52, leading to an F1 score of 0.67. While achieving a respectable recall of 0.85 for the Army Worm class, the precision is relatively lower at 0.76, resulting in an F1 score of 0.80. Similarly, the Bacterial Blight class shows a precision of 0.51, recall of 0.60, and F1 score of 0.55, indicating moderate performance. The model also did not perform well for the Target Spot class with 0.60 as precision, 0.85 as recall of 0.85, and 0.71 as F1 score. For the Powdery Mildew class, the VGG19 model demonstrates a high precision of 0.96 and an average recall of 0.77, resulting in an F1 score of 0.85. Lastly, the Healthy Leaves class shows good precision at 0.88 and recall at 0.83, with an F1 score of 0.85. Overall, while the model achieves high precision in some cases, it struggles with recall for several classes, impacting the F1 score and indicating room for improvement in its performance for certain categories.

Additionally, the computational time frames of various deep learning models have been also computed (**Table 7**) on applying to the cotton plant disease dataset. As, the system used for this study consists of a standard desktop architecture with an Intel i7 processor, 32GB of RAM, a 4GB GPU, and Windows 11 as the operating system. Hence, the training duration of each model varies depending on these computational requirements and the system’s hardware configuration. It has been found that EfficientNetB0 obtained the longest time of 10 hours and 56 minutes, closely followed by Inception V3 with 7 hours and 48 minutes. On the other, VGG19 took only 2 hours for the computation of the dataset while as moderate timings have been taken by ResNet50V2 and MobileNetV2 with 3 hours 1 minute and 4 hours 40 minute respectively. Apart from this, DenseNet169, Xception, and Hybrid (InceptionResNetV2) required relatively substantial training times which range from 6 to 8 hours. The models with complex architecture require longer computational times as compared to simpler architectures like VGG19.

**Table 7:** Computational time of models.

Models	Time Frame
**EfficientNetB0**	10 hr 56 min
**DenseNet169**	8 hr 5 min
**Xception**	9 hr 1 min
**MobileNet V2**	4 hr 40 min
**ResNet50 V2**	3 hr 1 min
**ResNet152 V2**	5 hr 5 min
**VGG19**	2 hr
**Inception V3**	7 hr 48 min
**EfficientNetB3**	5 hr 26 min
**Hybrid(InceptionResNetV2)**	6 hr 2 min

Bold values present the best values computed by the model.

Additionally, **Table 8** presents a comparative analysis of various studies focusing on the detection and classification of diseases affecting cotton plants using machine learning and deep learning techniques. Each study employs different datasets, classes of diseases, techniques, and their outcomes. Firstly, regarding the datasets used, researchers employ both customized datasets specific to cotton plants and publicly available datasets like CIFAR-10 (Canadian Institute for Advanced Research), cotton plant disease dataset (Rajasekar et al., 2021). The classes of diseases vary across studies, ranging from common issues like boll rot and fungal leafspot diseases to more specific diseases such as Cotton leaf curl virus and fusarium wilt. In terms of techniques, convolutional neural networks (CNN) are the most used, with variants like Tflite model, CoreML model (Kumar et al., 2022), and custom architectures like CottonLeafNet (Singh et al., 2023) and CoNet (Thivya Lakshmi et al., 2024) proposed in some studies. Additionally, models like ResNet50, InceptionVGG16, and DenseNet121 are also employed which showcase the diversity in model architectures. The outcomes, measured primarily in terms of accuracy, vary across studies i.e. from 90% to 99.96%. Notably, the highest accuracy is achieved by the current technique used in this paper i.e. the EfficientNetB3 architecture on a dataset encompassing Army Worm, Aphids, Target spot, Powdery Mildew, and Bacterial Blight.

**Table 8:** Analysis of the current work with the existing techniques.

Author’s Name	Dataset	Classes	Techniques	Accuracy
Kumar et al. (2022)	Customized dataset of cotton plants	boll rot and fungal leafspot diseases	CNN, Tflite model, CoreML model	90%
Memon et al. (2022)	Cotton dataset	Healthy, Leaf curl, Leaf spot, Verticillium Wilt, Nutrient Deficiency, Powdery Mildow, and Target Spot	CNN	98.53%
Singh et al. (2023)	Cotton Plant Disease dataset	Healthy, Blight, Soft Spots, Mottling etc.	Proposed CNN model (CottonLeafNet)	99.39%
Rajasekar et al. (2021)	CIFAR-10 dataset	–	ResNet50	98%
Naeem et al. (2023)	Cotton Plant Disease dataset	Fusarium wilt, Cotton leaf curl virus, bacterial blight	InceptionVGG16	98%
Kalaiselvi and Narmatha (2023)	Self captured dataset	Anthracnose, Bacterial blight, Cercospora leaf spot and Alternaria	fuzzy rough C-means + CNN	99%
Odukoya et al. (2023)	Fungal leaves captured using Digital camera	Healthy and unhealthy leaves	K Means and Support vector Machine	99.05%
Arathi and Dulhare (2023)	Cotton Plant disease dataset	Cotton leaf curl virus, bacterial blight, fusarium wilt	DenseNet121	91%
Hyder and Talpur (2024)	Cotton leaf disease dataset	Fussarium wilt, Bacterial blight, healthy leaves, and Curly virus	Proposed ML model	98.5%
Kukadiya et al. (2024)			Ensemble learning model (VGG16+ InceptionV3)	95%
Thivya Lakshmi et al. (2024)	Real time image data of cotton leaves	Unhealthy and healthy leaves	Proposed CNN (CoNet)	96%
Mohmmad et al. (2024)	Cotton Plant disease dataset	Aphids, Bacterial Blight, Curly Leaves, Powdery Mildew, and Verticillium Wilt.	VGG19	97.08%
**Our Study**	**Cotton Plant disease dataset**	**Army Worm, Aphids, Target spot, Powdery Mildew, and Bacterial Blight**	**Noise Removal, Contour Feature, Adaptive Thresholding, EfficientNetB3**	**99.96%**

Bold values present the best values computed by the model.

## 4 Discussion

The potential of deep learning techniques for detecting and classifying cotton plant diseases presents a promising avenue for addressing agricultural challenges on a global scale. However, realizing this potential requires careful consideration of various feasibility factors. Access to high-quality, labelled datasets is paramount, as it forms the foundation for training effective models, although obtaining such data may prove challenging, particularly for rare or localized diseases (Malar et al., 2021). Moreover, while deep learning models have demonstrated remarkable performance in image classification tasks, hence, to ensure their ability in detecting subtle symptoms and adapt to diverse environmental conditions remains critical. This necessitates robust model design and optimization strategies to enhance performance across different agricultural settings. Additionally, deep learning provides detailed insights into the severity as well as location of diseases within a field. By precisely mapping the distribution of diseases, farmers can adopt site-specific management practices, such as adjusted irrigation, targeted pesticide application, and optimized resource allocation. Moreover, the automation inherent as well as scalability of the deep learning models can also enable to efficiently monitor large agricultural areas using platforms like drones or satellites (Annabel et al., 2019). However, challenges such as the availability of diverse and representative datasets, the generalization of models to different environmental conditions, and the seamless integration of deep learning solutions into existing agricultural workflows require careful consideration and ongoing research to fully harness the potential of this technology in combating cotton plant diseases.

Apart from this, there are also several new improvements that can be done to enhance the current research and its practical applications. These improvements include (Askr et al., 2024; Woźniak and Ijaz, 2024):

- To enhance the performance of deep learning techniques for identifying and classifying cotton plant diseases, advanced optimization techniques such as learning rate scheduling, early stopping, and regularization methods should be fine-tuned.
- Integration of Multi-Modal Data where incorporation of additional data modalities such as spectral imaging, hyperspectral imaging, or thermal imaging alongside visual images can provide complementary information for disease detection. Fusion of multi-modal data can enhance the model’s ability to capture subtle disease symptoms and improve overall diagnostic accuracy.
- Explainable AI Techniques such as attention mechanisms, saliency maps, or feature visualization methods can help to interpret the decisions made by deep learning models. In fact, providing explanations for model predictions can increase trust and transparency in the disease detection system to facilitate better decision-making by end-users.
- Active Learning Strategies can optimize the data labelling process by selecting the most informative samples for annotation. By iteratively training the model on a small set of labelled data and actively acquiring labels for the most uncertain samples, the efficiency of the disease detection system can be enhanced while reducing labelling costs.
- Those models should be developed that are robust to environmental variability, such as changes in lighting conditions, camera angles, or plant growth stages, is crucial for real-world deployment. Adapting the models to diverse environmental conditions through data augmentation and domain adaptation techniques can ensure consistent performance in field settings.
- Design of scalable and deployable solutions that can be easily integrated into existing agricultural systems is essential for widespread adoption. Developing lightweight models optimized for edge devices, cloud-based solutions for centralized monitoring, and user-friendly interfaces for farmers can facilitate the practical implementation of disease detection technologies.

However, in the future work, there is significant scope to expand the current research on disease detection in cotton plants using deep learning techniques. It includes (Naga et al., 2024; Woźniak and Ijaz, 2024):

- Extension of the current applied models to handle a broader range of diseases and abnormalities that affect cotton plants. By incorporating additional classes into the classification system, the AI based system can provide more comprehensive insights for farmers and agronomists.
- To explore advanced transfer learning strategies to leverage pre-trained models effectively. Fine-tuning existing models or combining multiple models through ensemble techniques could enhance the overall performance and robustness of the disease detection system.
- Implementing more advanced data augmentation methods for increasing the size and diversity of the training dataset as it improves the ability of the model to work on unseen data with a good accuracy.
- To incorporate techniques such as model visualization or attention mechanisms which can help users in understanding how the model predicts the output?
- Developing a real-time monitoring system by integrating IoT and cloud technique to provide alerts to the farmers on continuously analysing the images of cotton plants so that they will be able to take decision promptly in saving the cotton plants.
- Building mobile applications for end user interaction where farmers can upload images of diseased plants to analyse and review recommendations for treatment. By this way, the system can adapt to local conditions and improve the accuracy of detecting and classifying cotton diseases on time.

Hence, in a nutshell it can be said that by incorporating these new improvements and to explore these avenues for future work into the research on disease detection in cotton plants, the field can advance towards more accurate, interpretable and user-centric solutions that address the challenges faced in sustainable agriculture and contribute to increased crop productivity and food security.

## 5 Conclusion

The research has shown the capability of artificial intelligence-based learning techniques to detect diseases in the leaves of cotton plants. The paper highlights the application of deep transfer learning approaches for their effective role in identifying and well as classifying various cotton plant diseases such as Target Spot, Bacterial Blight, Aphids, Army Worm, and Powdery Mildew. During the training of the models, it is recommended to prioritize EfficientNetB3 due to its superior performance as it achieved the highest accuracy of 99.96%. However, depending on specific needs such as computational constraints or model complexity, other models like MobileNetV2 or ResNet50V2 could also be considered, as they offer high accuracy with potentially lower computational overhead.

The paper, while showing promising results in detecting various diseases in cotton plants, also faces several limitations that need to be addressed. One major issue is the accurate generation of the region of interest (ROI) in the images. In plant disease detection, it is crucial to identify the specific areas affected by disease. If the ROI is not accurately detected, the model may analyse irrelevant parts of the image which will lead to misclassification and inaccurate predictions. Another limitation is the tendency of models to modelling error, particularly when training on fewer non-diverse datasets. Additionally, the computational complexity of deep learning models used in the paper is high, requiring substantial computational resources for both training and validation. This can be a bottleneck, especially when working with diverse datasets or deploying the model in resource-constrained environments. To mitigate these challenges, the use of optimization techniques, such as fine-tuning model parameters, is necessary to improve performance and reduce the risk of modeling errors. These limitations, if unaddressed, could impact the generalizability and robustness of the results, making it essential to refine the models and techniques used. Apart from this, looking ahead, the future scope of this research lies in enhancing the interpretability of deep learning models, exploring ensemble learning techniques to boost performance, and integrating real-time monitoring systems for proactive disease management in agriculture. By dealing with these challenges and incorporating new techniques, the field of disease detection in cotton plants can evolve to facilitate more efficient and sustainable agricultural practices. This proactive approach not only benefits crop yield and quality but also contributes to the overall resilience and productivity of agricultural systems.

## Data Availability

Publicly available datasets were analyzed in this study. This data can be found here: https://www.kaggle.com/datasets/dhamur/cotton-plant-disease.
